# Towards MnN as a replacement for IrMn

**DOI:** 10.1038/s41598-024-72886-y

**Published:** 2024-09-20

**Authors:** William Frost, Fatimah Alsaud, Robert A. Lawrence, Matt Probert, Gonzalo Vallejo Fernandez

**Affiliations:** School of Physics, Engineering and Technology, University of York, Heslington, YO10 5DD UK

**Keywords:** MnN, Antiferromagnetism, Exchange bias, Magnetic properties and materials, Spintronics, Condensed-matter physics, Materials for devices

## Abstract

There is an urgent need to identify new antiferromagnetic materials that offer a replacement for IrMn alloys for room temperature and above applications. This is driven by the scarcity and high cost of Ir. Recently, MnN with {002} texture grown on a Ta seed layer has been proposed as a cost-effective alternative. However, two key issues need to be addressed before this material can be considered a realistic alternative to IrMn: thick layers of approximately 30 nm are required due to its relatively low magnetocrystalline anisotropy and nitrogen diffusion into the Ta layer at relatively low temperatures results in poor temperature performance. In this work, we show a potential pathway to overcome these issues. By using a W rather than a Ta seed layer, N diffusion is minimized, if not eliminated, at temperatures exceeding $300\,^{\circ }\hbox {C}$. Furthermore, preferential {111} growth is achieved and a significantly enhanced anisotropy, $2.6 \cdot 10^{6}\,\hbox {erg}/\hbox {cm}^{3}$, has been measured. This value is almost identical to that measured for 3D randomly oriented IrMn.

## Introduction

The demand for faster, denser memory and data storage technology is constantly increasing, while the energy efficiency must also improve. Currently, the best technologies are based on ferromagnetic (*F*) storage layers in systems based on magnetic tunnelling junctions (MTJs). The most mature of these systems are the various iterations of magnetic random access memory (MRAM). However, recent developments have been suggested that MRAM and adjacent technologies could be based on antiferromagnetic (*AF*) materials. Such an avenue forms one part of the emergent field dubbed ‘antiferromagnetic spintronics’^1^. This is a promising route as the different timescales for spin dynamics of AF materials allow for faster switching. This is partly because the attempt frequency, $f_0$, of an *AF* material is several orders of magnitude higher than that of an *F* material, increasing from GHz ($1\cdot 10^{9}\,\hbox {s}^{-1}$) to THz ($1\cdot 10^{12}\,\hbox {s}^{-1}$)^2^. $f_0$ is an intrinsic property of a material, representing the rate at which the spin-moment attempts to reverse in the Néel-Arrhenius equation^3^

1 $$\begin{aligned} \tau = \frac{1}{f_0}\exp \left[ \frac{\Delta E}{k_BT}\right] \end{aligned}$$

where $\tau$ is the reversal lifetime and $\displaystyle \frac{\Delta E}{k_BT}$ is the ratio of the energy barrier to the reversal and the thermal energy of the system. It is immediately obvious from eq. (1) that any change in the value of $f_0$ can significantly affect the reversal time of a magnetic system and hence a memory device.

Modern, long-term data storage devices also universally utilise antiferromagnetic materials in the read-head of a hard disk drive (HDD). Every single single HDD produced contains a thin layer of antiferromagnetic material to exploit the exchange bias effect in an MTJ. Exchange bias is an additional energy term in a magnetic system that results in a shift in the magnetic hysteresis loop along the field axis when field annealed, offsetting the symmetry of the hysteresis loop from the $H_{app} = 0$ Oe line, where $H_{app}$ is the applied magnetic field^4^.

The current materials of choice for all of these applications are alloys of IrMn. These have excellent corrosion resistance and high magnetocrystalline anisotropy, *K*$$_{\textrm{AF}}$$, greater than $$4 \cdot 10^{7}\,\hbox {erg/cm}^{3}$$^5^, which leads to excellent thermal stability. Strong texturing of the {111} planes of the IrMn alloy in the sample plane is vital for the optimisation of these properties and for high values of the field shift or exchange bias, *H*$$_{\textrm{ex}}$$, of the order of several kOe^6^. This control is achieved through the use of seed layers, whereby different textures can be induced by different materials. For example, changing the seed layer from NiCr to Cu changes the texture of IrMn from a 2D to a 3D random texture^6^. However, this material relies on the availability of iridium. Iridium is mined as a small fraction of extracted platinum and contributes 0.03 parts per billion of the Earth’s crust. This has resulted in a dramatic increase in price from approximately $13k/kg in 2000 to approximately $163k/kg in 2021. Alternative materials such as PtMn still rely on the relatively expensive and rare platinum and requires high temperature annealing to induce the *AF* phase.

**Fig. 1:**
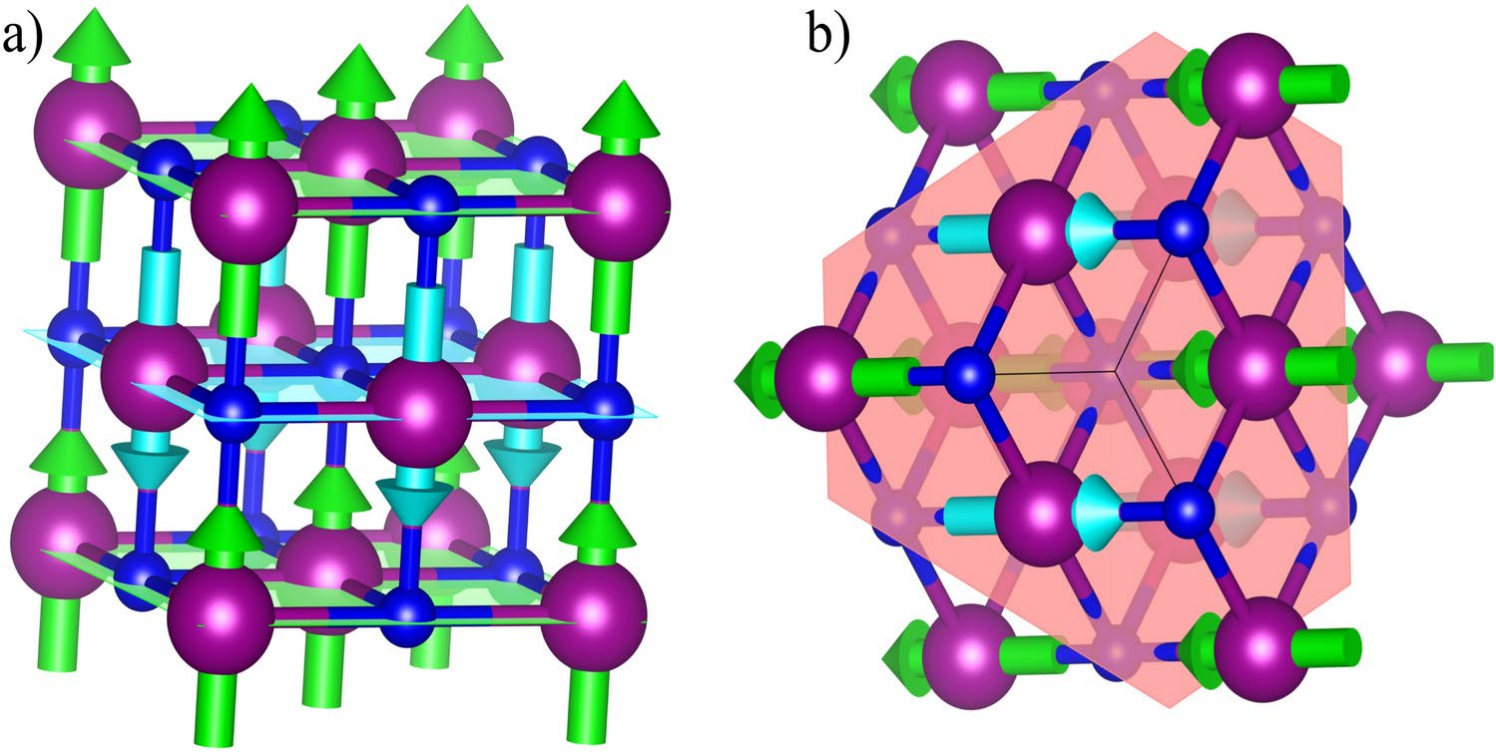
(**a**) Space and spin structure of bulk $\theta$-MnN (c/a = 0.95). Mn atoms are purple and N are dark blue. Arrows indicate the relative spin directions in the adjacent planes. (**b**) N-terminated {111} surface of $\theta$-MnN with the {111} plane highlighted in pink.

A promising alternative material is MnN, composed of two extremely abundant and cheap materials, both of which can be obtained with a low environmental impact. MnN has a complicated phase diagram^7^, but can exist in a quasi-equiatomic, antiferromagnetic $\theta$-phase as shown in Fig. 1(a). The bulk $\theta$-phase is tetragonal with a *c*/*a* ratio of 0.984, having the Mn spins oriented in alternating sheets in the $\left[ 001\right]$ direction. The significant work on the material has focused on the growth of MnN on seed layers of Ta. Works by Meinert et al.^8–10^ and Sinclair et al.^11^ have shown that this system can induce a positive strain in the plane of the MnN film, resulting in a *c*/*a* ratio of 1.04. This generates an effective anisotropy in the MnN of $\sim 1\cdot 10^{6}\,\hbox {erg/cm}^{3}$, which is not insignificant, but is substantially lower than that for the IrMn systems discussed above.

Additionally, the MnN layer is required to be very thick, approximately 30 nm. This is due to significant diffusion of N into the neighbouring Ta layer and therefore control of the stoichiometry such that the $\theta$-phase is maintained requires a large volume. As such, the material cannot be used in device applications where thin *AF* layers and repeatability of performance are key. For a recent review on this topic, please see Ref.^12^.

In this work we propose a new route to potentially improve the viability of MnN by using tungsten (W) as a seed layer. We show experimentally and via density functional theory (DFT) calculations that W is a superior buffer to the diffusion of N compared to Ta. Critically, by using this seed layer we can induce a {111} texture in the MnN layer which may increase the spin coupling to the *F* layer, as shown in Fig. 1(b), where the preferred spin structure is parallel to the alignment of the *F* layer. This opens promising avenues for the development of MnN as a viable *AF* material that is both cheaper and more sustainable in the long term.

## Results

### Modelling of nitrogen diffusion

The diffusion of N into a seed layer is controlled by the chemical kinetics of diffusion, governed by the Arrhenius equation

2 $$\begin{aligned} k = Ae^{\frac{-E_a}{k_BT}} \end{aligned}$$

where *k* is the reaction rate constant, $E_a$ is the activation energy, $k_BT$ is the thermal energy, and *A* is a system-dependent constant. Calculating the parameters for the Arrhenius equation, such as the activation energy $E_a$, is extremely costly within *ab initio* theory, especially for diffusion within a solid. In this work, we have instead calculated the enthalpy changes of the system before and after diffusion, and then used Hammond’s postulate^13^ in conjunction with the Bell-Evans-Polanyi (BEP) principle^14^ to predict the nature of the barrier (early, intermediate or late)^13^ and infer the impact this has on the kinetics.

The BEP principle may be broadly stated as *for a given family of reactions, there exists a linear relationship between the activation energy and the change in enthalpy of the reaction*. Since the diffusion of N into a transition metal from MnN is clearly the same “family”, we may then infer that the greater change in enthalpy will give a lower activation energy for the barrier.

Density functional theory within the local density approximation (LDA)^15^ as implemented within the plane-wave pseudopotential code CASTEP^16^ was used to simulate the total energy and structure of a MnN bilayer, passivated with hydrogen, on both a Ta and a W seed layer. For both seed layers a single N ion was repositioned to an interstitial site in the metallic substrate and the structure was re-relaxed. Spin was treated as collinear, and initialised to 5 $\mu _B$ per Mn ion, whilst being allowed to relax during the self-consistent field (SCF) process.

An 1100 eV cut-off energy was used with the CASTEP C19 pseudopotential library, with the total energy converged to $1 \cdot 10^{-7}\,\hbox {eV/atom}$. The structures were relaxed to better than 0.075 eV/Å, and a total change in energy of less than $5\,{\upmu }\hbox {eV/ion}$. The in-plane lattice constants of the respective seed layer were kept fixed to a previously optimised value for the bulk seed layer in order to model the effects of growing a thin layer of MnN on top of a layer of Ta or W. Finally, a vacuum gap of at least 12 Å, was used with H-passivation in order to minimize interaction between neighbouring images and minimise surface reconstruction due to dangling bonds.

**Fig. 2:**
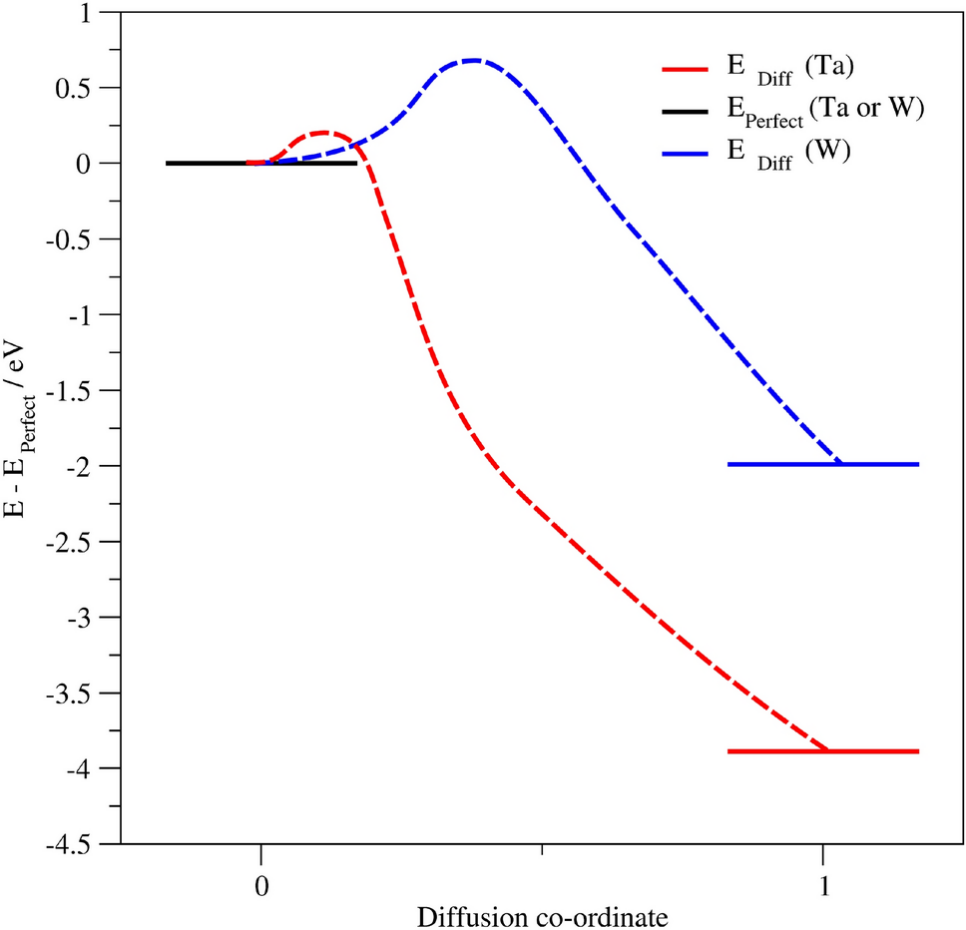
Enthalpies for the diffusion of N from MnN into a substrate made of either Ta or W, normalised such that the energy before diffusion is 0 eV. Blue (W) and red (Ta) dotted lines indicate an approximation of the relative transition state barrier positions as might be expected from the Bell-Evans-Polanyi principle in order to guide the eye, and there is no quantitative significance to the turning point shown.

For diffusion of a single atom of N into Ta, the change in enthalpy is $-3.89$ eV, whereas this value is $-1.88$ eV for N diffusion into W, as shown in Fig. 2. This indicates that the energy barrier against diffusion should be lower for Ta than W.

One may also use Hammond’s postulate^13^ (that similar structures have a similar energy) to comment on the nature of the barrier, which are usually categorised as *early*, *intermediate*, or *late*. For a low-barrier exothermic reaction, one expects to see an early barrier, whereas a higher barrier or less exothermic reaction will have more intermediate properties.

According to Polanyi^17^, in the presence of an early barrier (i.e. for a significantly exothermic reaction) the reaction rate may be increased by increasing the initial translational velocity of the incoming particle - which is precisely the conditions that one may expect to see in growth by sputtering. This suggests that the sputtering process onto Ta is highly likely to lead to diffusion of N into the Ta, whereas the N is more likely to remain at the surface of the W due to the more intermediate nature of the barrier. Hence, this suggests that W could be more resistant to N diffusion than Ta, potentially making it a better seed layer. Further details on the DFT calculations are available in the supplementary information.

### Crystallography of as-deposited samples

**Fig. 3:**
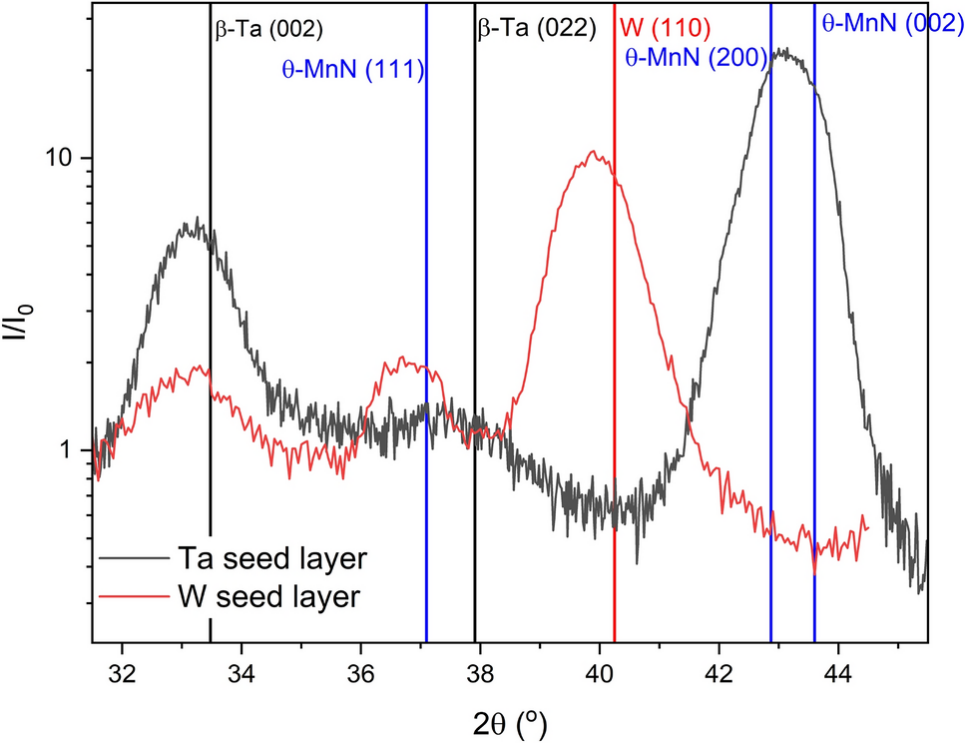
Symmetric, out-of-plane $\theta -2\theta$ scans for the samples with seed layers of W and Ta, showing the different growth directions of $\theta$-MnN, with bulk positions indicated.

Figure 3 shows the out-of-plane, symmetric $\theta -2\theta$ scans for samples of 28 nm of MnN deposited on 10 nm thick Ta and W seed layers. The first thing that is clear is the different growth directions of the MnN on the two seed layers. For the sample with the seed layer of Ta, as expected there is a $\langle 100\rangle$ growth direction to the MnN, as shown previously^9^. However, in previous studies the Ta is assumed to have grown in the $\alpha$-Ta phase. Here the $\beta$-Ta phase has formed with a preference for the long *c* axis perpendicular to the substrate indicated by the (002) reflection. This reflection is present in the sample with a W seed layer due to the presence of a Ta capping layer. For the seed layer of W, however, the MnN $\langle 100\rangle$ reflections do not appear and have been replaced with a broad {111} reflection. The reflection could be broad for several reasons, but is most likely due to the lattice parameter of MnN being very sensitive to any variation in composition and having a broad random distribution.^7^

This change of growth direction to have {111} planes perpendicular to the sample normal is of great interest as it will increase the spin coupling to the *F* layer compared to conventional growth which results in orthogonal spin alignment. If the *AF* ordering of the MnN can be set along these planes, it may lead to an improvement in the properties of the material, most notably the value of $K_{AF}$ and hence to thermal stability. This is discussed further in the Magnetic Measurements section.

**Fig. 4:**
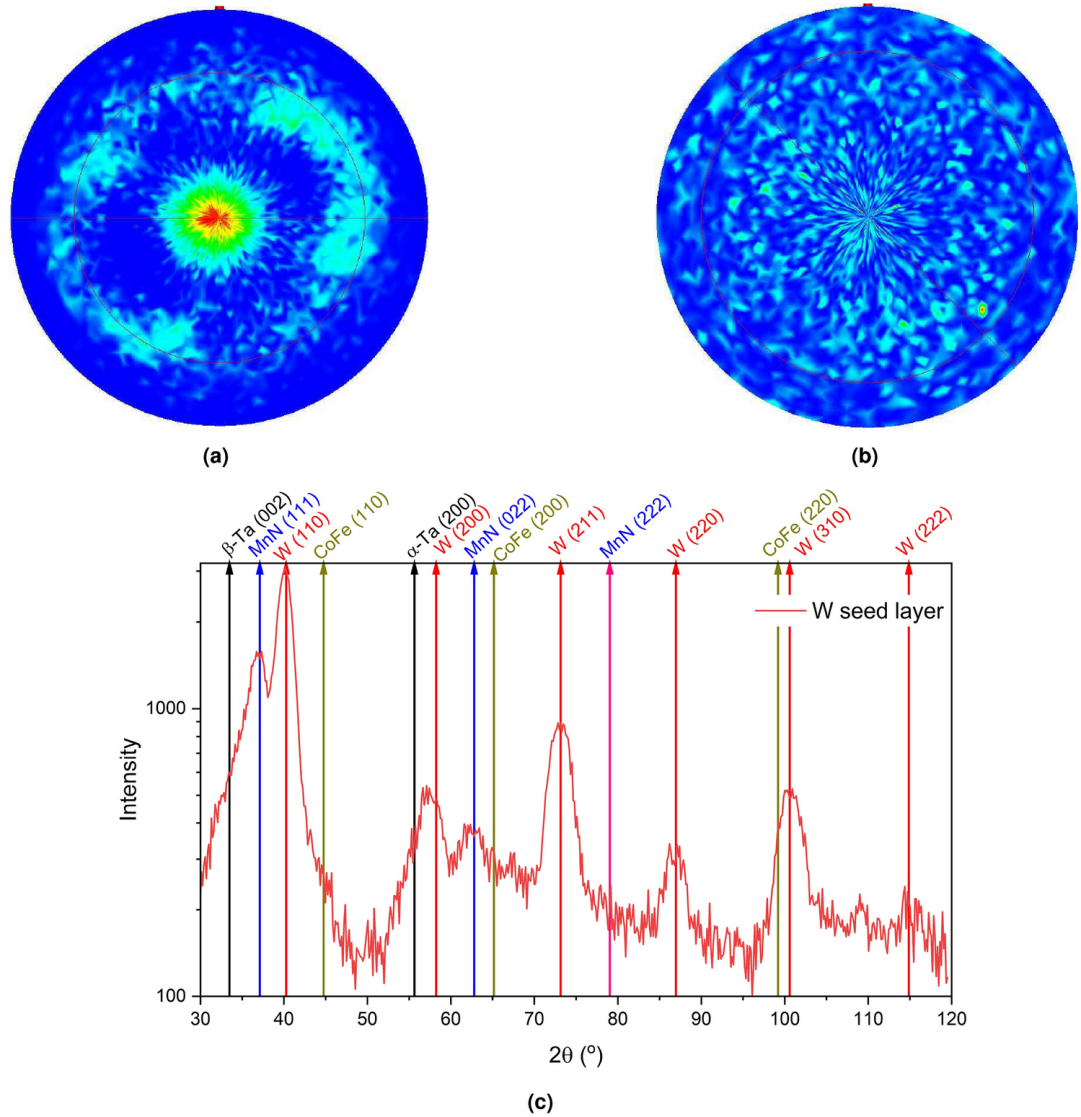
Pole figure scans of the (**a**) W{110} reflection and (**b**) the MnN{111} reflection for the sample with a seed layer of W. The W layer is slightly textured with a clear ring around ${30}^{\circ }$, whereas the MnN is almost totally 3D random. (**c**) Asymmetric, grazing incidence out-of-plane $2\theta$ scan for the sample with a seed layer of W, showing the polycrystalline growth directions of W, with bulk positions indicated.

Pole figure scans were performed on the out-of-plane reflections of the W and MnN layers and are shown in Fig. 4. For the {110} reflection of the W layer shown in Fig. 4a, there is a central maximum and a ring observed around ${30}^{\circ }$, indicating texturing of the {110} planes. The diffuse nature of the ring and broadness of the rocking curves indicate that the texturing is very weak. For the MnN reflection shown in Fig. 4b, the level of texturing is removed and the {111} reflection is almost totally 3D random. Any preference observed in the symmetric scans in Fig. 3 is extremely weak.

Figure 4c shows the grazing-incidence, asymmetric $2\theta$ scan for the sample with a seed layer of W. The major reflections of the W layer are all present indicating an almost 3D-random, polycrystalline growth of the W seed layer with a diffraction pattern resembling that of a random powder. The MnN{111} reflection is also still visible in the grazing geometry, showing that the MnN layer is growing without any strong texture. Further reflections from the MnN such as the {002} may also be hidden by the intensity of the W {110} peak. These data agree with the observations of the pole figure scans in Fig. 4 and the rocking curve analysis. However, it is useful to know that the layers are crystalline despite the lack of texture in the films. Further improvement of the properties of the multilayer can potentially be achieved by improving the the texture of the seed layer or the MnN layer. This opens up exciting potential for future work to investigate the effects of heated depositions and further optimisations of growth parameters to maximize the *K*$_{\textrm{AF}}$.

**Fig. 5:**
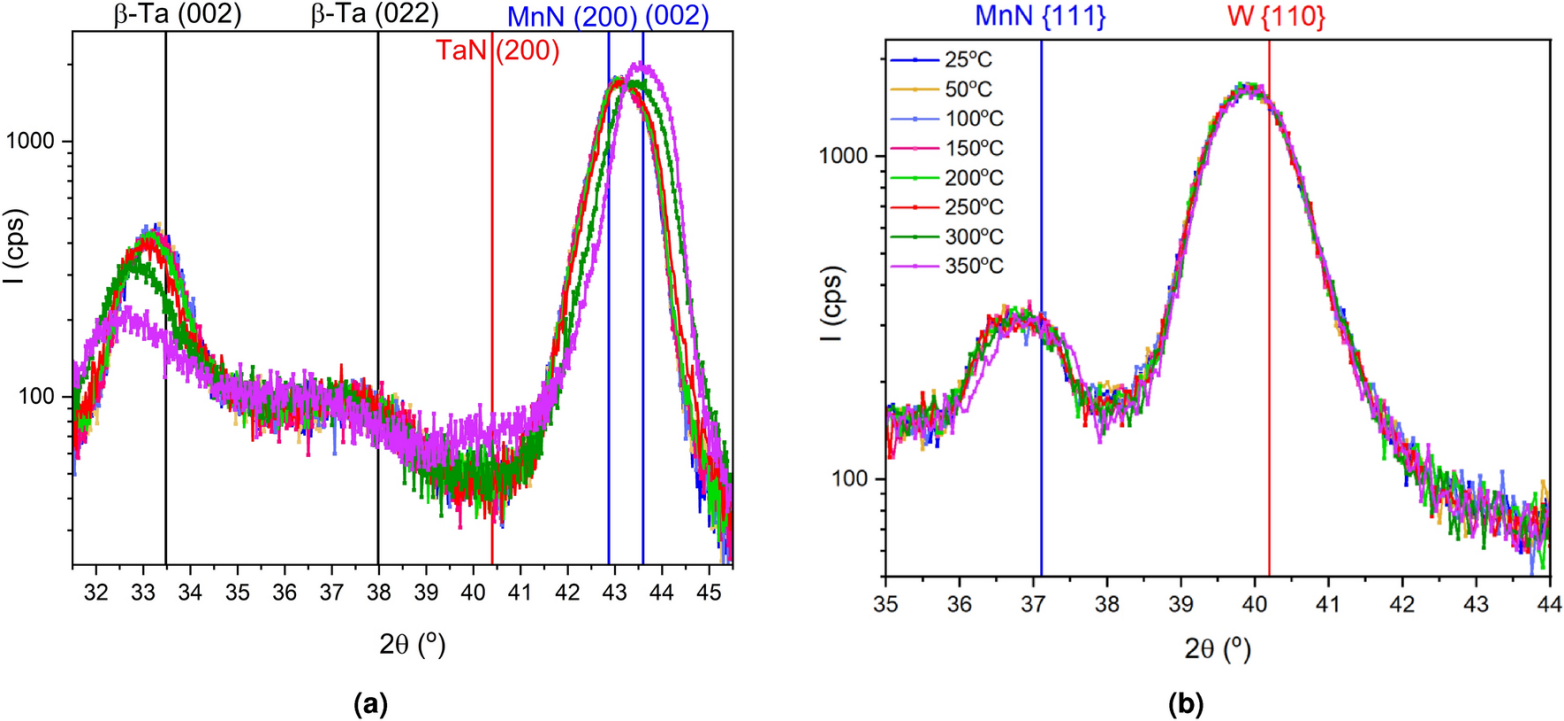
Symmetric, out-of-plane $\theta -2\theta$ scans for the samples with a seed layer of (**a**) Ta and (**b**) W with increasing temperature. Samples were heated to the indicated temperature for one hour and then XRD data were taken at $25\,^{\circ }\hbox {C}$.

### Annealing effects

Figure 5 shows the symmetric, out-of-plane $\theta -2\theta$ scans for the samples as a function of annealing temperature. The samples were heated *in situ* under vacuum for one hour and then cooled to $25\,^{\circ }\hbox {C}$ for measurement in order to remove any thermal expansion effects. Figure 5a shows the data for the sample with a seed layer of Ta. The thermal instability of the system is immediately obvious as the positions and intensities of the $\beta$-Ta (002) and MnN {002} reflections are affected by small increases in temperature to $200\,^{\circ }\hbox {C}$. The substantial shift from the bulk reflections for $\beta$-Ta indicate a large change in lattice parameter, potentially caused by the formation of TaN due to the ease of diffusion of N into Ta. This is also seen in the appearance of a weak reflection around $40.5^{\circ }$, potentially due to the face-centred-cubic (fcc) TaN {200} reflection.

The Ta scan result is counterposed by the same experiment shown in Fig. 5b for the seed layer of W. Here, there is no observed crystallographic change up to $300\,^{\circ }\hbox {C}$, and only a small shift in the peak position of the MnN {111} reflection by $0.1^{\circ }$ in $2\theta$, towards the bulk location indicating a reduction in the strain in the lattice with thermal annealing. The peak shape of the MnN {111} reflection is extremely broad and square, indicating a range of strains. There are also potentially composition variations which in turn lead to further lattice constant distributions. However, the thermal stability of the system is far superior to that of the Ta-based system as will be shown below. A significant improvement in thermal stability is ideal for applications such as the automotive industry, where device temperatures can often exceed $180^{\circ }\hbox {C}$. This is further discussed in the next section with regards to a significant impact on high temperature magnetic measurements where thermal stability is crucial.

### Magnetic measurements

**Fig. 6:**
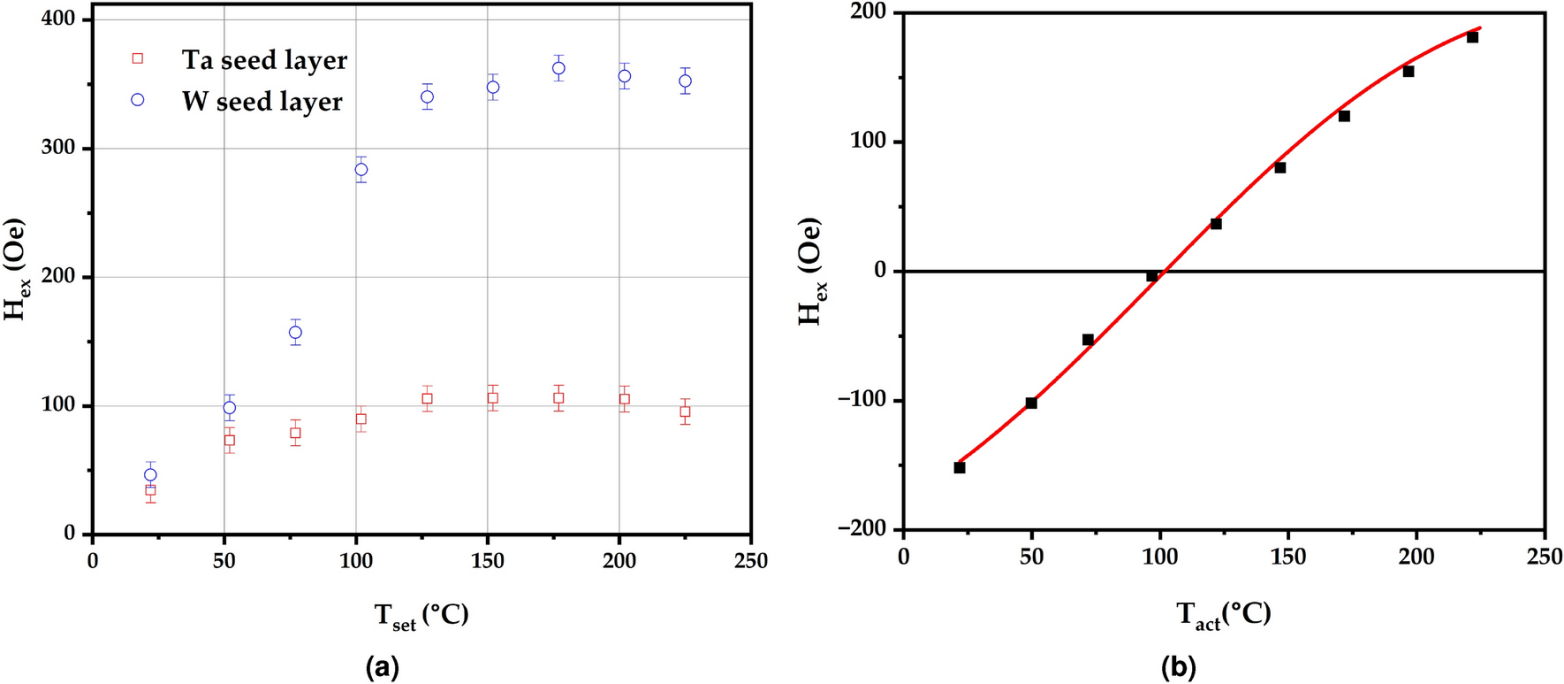
(**a**) The variation of the loop shift, *H*$_{\textrm{ex}}$, as a function of the setting temperature and (**b**) the blocking temperature distribution for the sample deposited on a W seed layer.

Figure 6a shows the variation of the shift of the hysteresis loop as a function of the setting temperature $T_{set}$ for the samples deposited on both W and Ta seed layers. The samples were heated to a temperature $T_{set}$ for 60 minutes in the presence of a 10 kOe magnetic field and subsequently cooled down to room temperature. A hysteresis loop was then measured. The maximum value of $T_{set}$ was $225~^{\circ }$ C as that was the highest temperature that could be used without damaging our VSM heater. In both cases, as the value of $T_{set}$ is increased, there is a sharp increase in the measured value of the loop shift. This is due to the gradual setting of the *AF* grains as the setting temperature is raised, but still well below the bulk Néel temperature of MnN at $650\,^{\circ }\hbox {C}$. The data for the samples with a W seed layer indicate that the whole system can be set at temperatures $\ge 150~^{\circ }$ C without showing any deterioration in the magnetic properties at higher temperatures. On the other hand the magnitude of the loop shift for the sample deposited on a Ta seed layer seems to saturate at temperature $\ge 125~^{\circ }$ C. However, it appears that at $225~^{\circ }$ C the exchange bias is starting to decrease. This would agree with the XRD data shown in Fig. 5 which shows the thermal sensitivity of the Ta based system up to $350~^{\circ }$ C. This is also consisitent with previous work by Meinert et al.^8^, suggesting that W provides a better barrier against nitrogen diffusion as predicted by DFT.

The question then arises as to whether this will result in different thermal stability as a function of the different seed layer materials. Figure 6b shows the distribution of blocking temperatures for the sample deposited on a W seed layer. The solid line is a calculated fit to an experimental blocking curve and the details of how the fitting was performed can be found in the supplementary information. The temperature at which $H_{ex}$ reaches 0 is known as the median blocking temperature and is the temperature at which equal volume fractions of the *AF* grains are oriented in opposite directions. In order to obtain the curve shown in Fig. 6b the sample is subject to thermal activation with the CoFe layer saturated in the negative direction at increasing values of an activation temperature $T_{act}$. By varying $T_{act}$ the distribution of energy barriers to reversal in the *AF* layer can be mapped and allows for the determination of the effective anisotropy constant of the material $K_{AF}$. As described in the supplementary information, the fit in Fig. 6b yields a value of the anisotropy constant at room temperature for MnN grown on W of $2.6 \cdot 10^{6}\,\hbox {erg/cm}^{3}$. This is significantly larger than measurements of MnN when grown using the same deposition system on a Ta seed layer but still an order of magnitude lower than the maximum value reported for IrMn^5^. However, the latter was measured in a sample with strong in-plane {111} texture. For a system with 3D random texture, as is the case for the sample described in this work, a significant lower value of $\sim 2.8 \cdot 10^{6}\,\hbox {erg/cm}^{3}$ was reported^6^. This suggests that controlling the texturing of IrMn, and hence the coupling to the *FM*, can result in an increase in the effective anisotropy constant of the material by a factor $\sim$10. If that were also true for MnN, values in excess of $1\cdot 10^{7}\,\hbox {erg/cm}^{3}$ may be possible, highlighting the potential of MnN as a replacement for IrMn.

Comparing Fig. 6a and b it is apparent that the values of the loop shift in the former for values of $$T_{set}$$$$\ge$$$$150~^{\circ }$$ C are much higher than the value observed when thermally activating at $$25~^{\circ }$$ C. This is due to the presence of a very strong training effect, i.e. decrease in the loop shift on repeated field cycling, which is removed during the blocking temperature measurement. This is consistent with data for IrMn where strong training is present in systems with 3D random texture. Interestingly, for IrMn training is removed when there is strong in-plane texture^18^.

The sample deposited on Ta also displays a very strong training effect. Once training is removed, the value of the loop shift is very small, <10 Oe. This makes the determination of the anisotropy constant difficult as any offset in the field, even if small, will have a significant impact on the exact value of the blocking temperature which would affect the measured value of the anisotropy constant. For this reason, we have not attempted to estimate a value of *K*$_{\textrm{AF}}$  for the sample deposited on Ta. However, it is evident that the effective anisotropy in this sample is very low.

## Conclusions and future work

In this paper, we have shown that MnN grown on a W seed layer is a promising candidate to replace IrMn alloys in high performance spintronic devices. When replacing the commonly-used seed layer of Ta, the W induces a {111} texture, albeit weakly, in the MnN. This change in texture is reflected by a significant increase in the effective anisotropy constant of the material. A value of $2.6 \cdot 10^{6}\,\hbox {erg/cm}^{3}$ is obtained at room temperature which is similar to the value obtained for {111} 3D randomly oriented IrMn^6^. Furthermore annealing data from XRD show that the W system is resilient to high temperatures ($\ge 300~^{\circ }$ C) as would be seen in applications such as the automotive industry. This opens up exciting opportunities for future work on W/MnN systems to improve their texture and subsequently their effective anisotropy and thermal properties.

## Methods

Samples were deposited using a High Target Utilisation Sputtering System (HiTUS) at room temperature on Si (001) substrates. The substrates are exposed to the plasma before deposition to ensure a clean and oxide free surface for deposition. The deposition pressure was optimised and set at 1.8 mTorr while the gas composition was set at 75:25 Ar:N$_2$ to achieve the desired, quasi-equiatomic composition. For more information please see the supplementary information. Samples were deposited with the structure Si(001)//*X* (15)/MnN (28)/Co$_{70}$Fe$_{30}$ (2.5)/Ta (5) where *X* is the seed layer of Ta or W and the thicknesses are given in nm in brackets. The F CoFe layer is used as the sensing layer to probe the effective value of the anisotropy and magnetic behaviour of the system.

Crystallographic characterisation was undertaken using a Rigaku SmartLab XRD, with a 9kW rotating anode and a Ge (220) monochromator for an angular precision of $0.01^{\circ }$. A six axis goniometer was used for rotational measurements and allows for the measurement of pole figure scans. An Anton-Paar DHS1100 furnace was used for in-situ annealing of samples up to $350\,^{\circ }\hbox {C}$ in a vacuum of $5 \cdot 10^{-2}\,\hbox {mbar}$. The magnetic measurements were performed using a MicroSense Model 10 VSM with a field precision of 1 Oe and a sensitivity of $1 \cdot 10^{-6}\,\hbox {emu}$. The temperature was controlled to within $4\,^{\circ }$C with a variation of $<0.1\,^{\circ }$C over the measurement period.

## Supplementary Information

Supplementary Information.

## Data Availability

The data that support the findings of this study are available from the corresponding authors upon reasonable request.
