# Supplementary material for: Towards MnN as a replacement for IrMn
Source: Sci Rep. 2024 Sep 20;14:21944. doi: 10.1038/s41598-024-72886-y (PMC11415361; doi:10.1038/s41598-024-72886-y)
Supplement: Supplementary file 1 — Supplementary Information. [file 41598_2024_72886_MOESM1_ESM.pdf]

Supporting information: Towards MnN as a replacement for IrMn

William Frost,* Fatimah Alsaud, Robert Lawrence, Matt Probert, and Gonzalo Vallejo-Fernandez*

School of Physics, Engineering and Technology, University of York, Heslington, YO10 5DD, UK

E-mail: william.frost@york.ac.uk; gonzalo.vallejofernandez@york.ac.uk

S-1: Details of DFT simulations of MnN on Ta and W

Initially, the stacking order of bulk MnN on a $\langle 111 \rangle$ substrate was investigated, and it was found that for both W and Ta it was preferable to have the N stacked on top of the substrate ions rather than the Mn. The simulation cells are shown in supplementary fig. 1. The H-passivation layer induced a slight buckling to the top MnN layer, which might be expected to have a slight effect on the magnetic ordering within the system, but not on the diffusion calculations which are the primary focus of this work. The W substrate also induced buckling on the *lower* layer of MnN, which was not present for the Ta substrate, which might be indicative of a stronger bonding interaction for the W-MnN than the Ta-MnN.

These bilayers exhibit a notable reduction in spin compared with an infinite bulk crystal of MnN. These results are summarised in supplementary table 1. We note that the spin moments in the upper bilayer are suppressed more than for the lower bilayer. Some of this (approximately 0.5 μ_B) is due to charge transfer from the H (which is electron-donating) filling some of the orbitals in the Mn and causing spin pairing, thereby reducing the net moment. This is the only cause to change of spin moment for the bilayer on Ta.

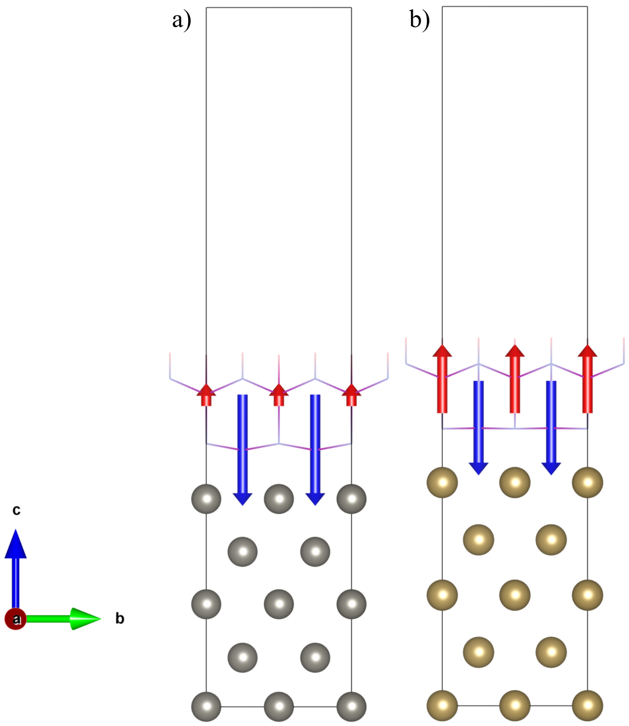

Supp

Supplementary Figure 1: Simulation cells used for this project. The seed layer is a) W and b) Ta, which are shown by grey and brown balls respectively. On top of each the MnN bilayer and the H-passivation layer are represented by a stick diagram, with arrows representing the spin magnitude.

For the bilayer on W, however, a notable imbalance between the spin magnitudes present in the upper and lower layer is present. This may be attributable to the significant difference in the Pauling electronegativities of the atoms involved; 1.55 for Mn, 1.5 for Ta, 2.36 for W and 3.04 for N. This indicates that whilst electrons have a weak preference for being near a Mn rather than a Ta atom, they have a fairly strong preference for being near a W rather than a Mn atom.

Supplementary Table 1: Spin moments on Mn by layer in the H-passivated cells without diffusion and for Bulk MnN. Note that the presence of the interfaces lead to a significant reduction in the spin-moments present on the Mn ions, which is especially noticeable for the W substrate.

Substrate	Spin Moment/ μ_B	
	1st Layer	2nd Layer
None (Bulk)	-3.55	3.55
Ta	-1.83	1.34
W	-2.15	0.45

The effects this redistribution of charge might be expected to have on the magnetocrystalline anisotropy cannot be taken from these collinear calculations, nor can the changes that might be

induced by a thicker layer of MnN, however it strongly suggests that interfacial effects may not be simply dismissed for device stacks such as these.

S-2: Process gas mixture optimisation

The nitrogen content of the process gas was varied to investigate the effects on the magnetic properties of MnN on seed layers of Ta and W. The samples were set in a field of 10 kOe for half an hour and the value of exchange bias taken as a measure of the quality of the sample. A setting temperature of 150 °C was used to avoid Nitrogen diffusion. As shown in supplementary fig. 2 a maximum is observed around (70–80) % and as such a gas mixture of 75% N_2 was used for all depositions of films presented in the paper.

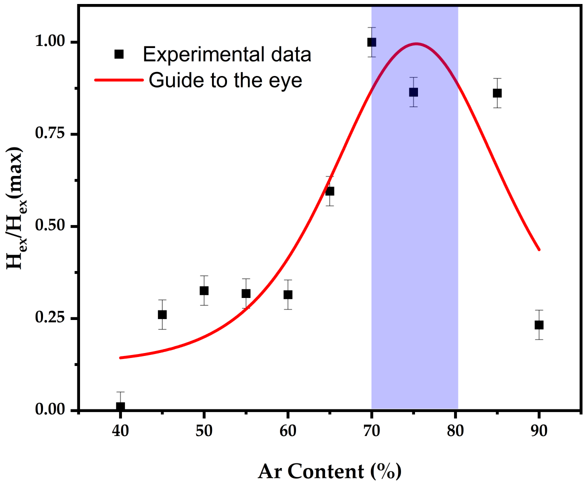

Supplementary Figure 2: The variation in the exchange bias compared to the maximum obtained value as a function of the nitrogen content used in sputtering, showing a peak around 75% N_2:Ar.

S-3: Process pressure optimisation

The process pressure was varied to investigate the effects on the magnetic properties of MnN on seed layers of Ta and W. The samples were set in a field of 10 kOe for half an hour and the value of exchange bias taken as a measure of the quality of the sample. Again a setting temperature of

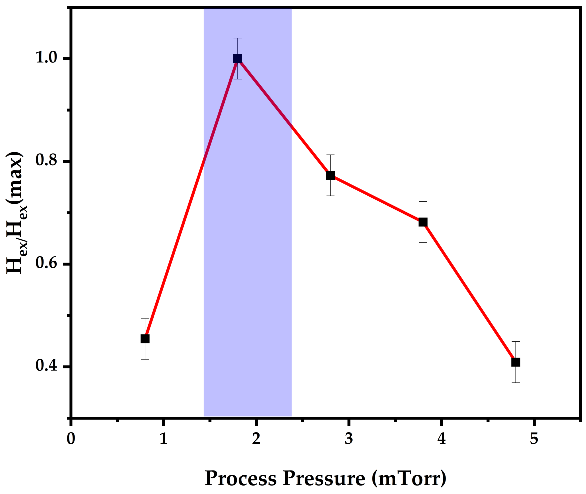

Supplementary Figure 3: The variation in the exchange bias compared to the maximum obtained value as a function of the process pressure used in sputtering, showing a peak around 1.8 mTorr

150 °C was used. As shown in supplementary fig. 3 a maximum is observed at 1.8 mTorr and as such this process pressure was used for all depositions of films presented in the paper.

S-4: Hysteresis loops after setting

Supplementary Figure 4 shows the room temperature hysteresis loops for the samples deposited on (a) a Ta and (b) a W seed layer after setting for an hour at different temperatures in a 10 kOe applied magnetic field. In the case of the sample deposited on W, supplementary fig. 4(b), the squareness of the hysteresis loops increases with increasing setting temperature with no detriment to the magnitude of the loop shift at the highest setting temperature. This suggests that any diffusion processes that might have taken place during the heat treatment are insignificant. The trend is pretty similar for the sample deposited on Ta, supplementary fig. 4(a), although at 225°C the loop shift is slightly lower than that measured after setting at ∼200°C. This suggests that small changes might have happened in the microstructure of the sample after the last round of annealing at 225 °C. This agrees with the X-ray diffraction data presented in the main manuscript.

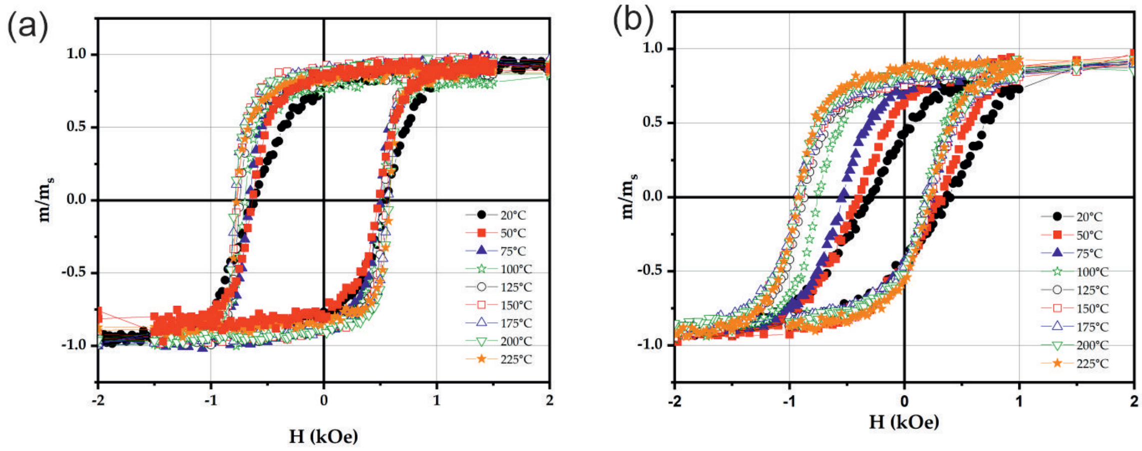

Supplementary Figure 4: Room temperature hysteresis loops measured after setting for the (a) Ta and (b) W samples.

S-5: In-plane TEM micrographs and grain size analysis of MnN

Supplementary Figure 5 shows bright field transmission electron micrographs taken in plane to determine the lognormal grain size distributions of the two systems. Samples with composition (Ta or W)(15 nm)/MnN(28 nm)/CoFe(2.5 nm)/Ta(10 nm) were deposited on TEM grids. In previous work we have shown that the grain size of samples deposited on Si substrates and TEM grids is the same due to the large separation, 30 cm, between the target and the substrates in our sputtering system.[1] The samples were grown using the optimised conditions described earlier. Both images are taken at x100k magnification for direct comparison. By comparing the two images it is immediately obvious that the grain size in the Ta sample is much larger. By analysing over 500 grains for each sample using an equivalent area method, it was found that the two systems have median grain diameters of (13.4±0.5) nm and (6.5±0.4) with standard deviations of (0.32±0.02) and (0.28±0.02) for Ta and W, respectively. Note that the standard deviations quoted correspond to the standard deviation of the respective lognormal distribution and, hence, have no units. The distributions for both samples are shown in supplementary fig. 5(c) and supplementary fig. 5(d) for Ta and W, respectively.

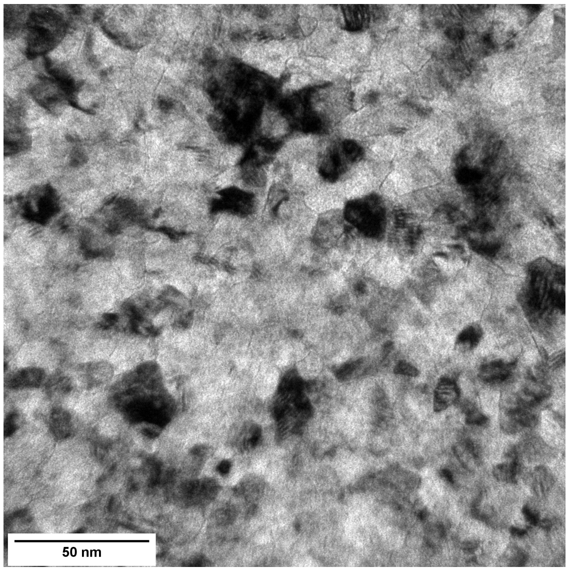

(a)

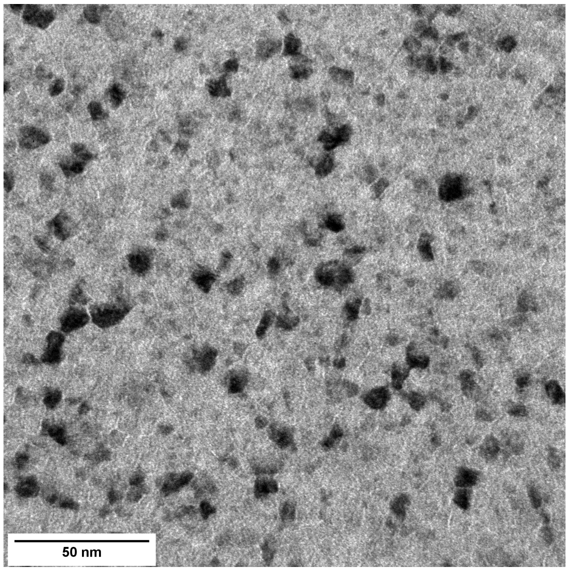

(b)

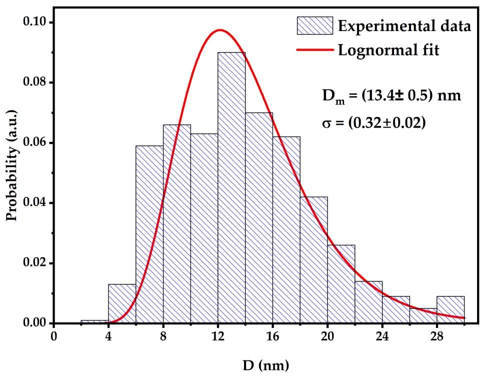

(c)

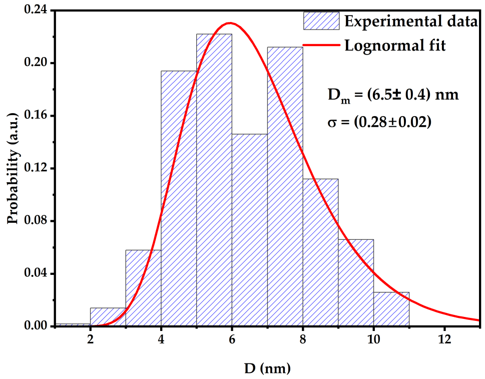

(d)

Supplementary Figure 5: Transmission electron micrographs, in-plane, of the (a) Ta and (b) W based systems. Both images are taken at x100k magnification in the bright field condition. The associated grain size distributions calculated from 500 grains are shown in (c) and (d) for the Ta and W based samples, respectively.

S-6: Blocking temperature distribution measurement and fitting

The method to measure the distribution of blocking temperatures in exchange bias systems has been described in detail previously.[2] The samples were first annealed for 90 minutes in the presence of a 10 kOe magnetic field at a setting temperature T_{set} = 225 °C. The system was then cooled to room temperature and the applied field reversed so that the ferromagnetic layer was saturated in

the opposite direction. The temperature was then raised to an activation temperature T_{act} and held in this configuration for 30 minutes. The samples were subsequently cooled back to room temperature and a hysteresis loop measured at that temperature after cycling the applied field to remove the training effect. The value of the loop shift $H_{\mathrm{ex}}(T_{act})$ at any given activation temperature T_{act} is given by

$$H_{ex}(T_{act}) \propto -\int_{V_c(T_{meas})}^{V_{act}(T_{act})} f(V)\,dV + \int_{V_{act}(T_{act})}^{V_{set}(T_{set})} f(V)\,dV \tag{1}$$

where $V_c(T_{meas})$ is the critical volume that is thermally stable at the temperature of measurement, $V_{act}(T_{act})$ is the critical volume being activated at the activation temperature T_{act} and $V_{set}(T_{set})$ is the largest volume that can be set during the field setting process. $f(V)$ is the distribution of energy barriers in the AF layer which is controlled by both the distribution of grain volumes in the AF layer and the anisotropy constant of the material K_{AF}. Assuming K_{AF} is uniform across grains, the distribution of energy barriers reduces to the distribution of volumes in the system. The values of V_c, V_{act} and V_{set} can be calculated as follows

$$V_c(T_{meas}) = \frac{ln(t_m f_0) k_B T_{meas}}{K_{AF}(T_{meas})} \tag{2}$$

$$V_{act}(T_{act}) = \frac{ln(t_{act} f_0) k_B T_{act}}{K_{AF}(T_{act})} \tag{3}$$

$$V_{set}(T_{set}) = \frac{ln(t_{set} f_0) k_B T_{set}}{K_{AF}(T_{set})} \tag{4}$$

where K_{AF} is the anisotropy constant of the MnN layer and f_0 is the attempt frequency of the material. The parameter t_m in eq. (2) refers to the measurement time. Similarly, the factors t_{act} and t_{set} in eq. (3) and eq. (4) refer to the time used for the thermal activation and setting processes, respectively. For our simulations t_m = 1.0 s, t_{act} = 1800 s and t_{act} = 5400 s as those were the values used in the experiments. Similarly T_{meas} = 25 °C, T_{set} = 225 °C and 25$\leq T_{act} \leq$ 225 °C. Finally,

the temperature dependence of the anisotropy was taken as[2]

$$K(T) = K(0)\left(1 - \frac{T}{T_N}\right) \tag{5}$$

where $K(0)$ is the value of the anisotropy constant at absolute zero and T_N is the Néel temperature of the material. In our case T_N was taken as ∼380 °C.[3] Using the grain size data shown in Section S-4 and the parameters mentioned above, the fit shown in fig. 7 of the main manuscript was obtained. The value of the attempt frequency was taken as $f_0 = 1.4 \cdot 10^{12}\,\mathrm{s}^{-1}$.[4] This yielded a value of the anisotropy constant for MnN at room temperature of $2.6 \cdot 10^6\,\mathrm{erg/cm}^3$.

References

(1) Vopsaroiu, M.; Fernandez, G. V.; Thwaites, M. J.; Anguita, J.; Grundy, P. J.; O'Grady, K. Deposition of polycrystalline thin films with controlled grain size. *Journal of Physics D: Applied Physics* **2005**, *38*, 490.

(2) O'Grady, K.; Fernandez-Outon, L.; Vallejo-Fernandez, G. A new paradigm for exchange bias in polycrystalline thin films. *Journal of Magnetism and Magnetic Materials* **2010**, *322*, 883–899.

(3) Leineweber, A.; Niewa, R.; Jacobs, H.; Kochelmann, W. The manganese nitrides η-Mn_3N_2 and θ-Mn_6N_{5+x}: Nuclear and magnetic structures. *Journal of Materials Chemistry* **2000**, *10*, 2827–2834.

(4) Alsaud, F.; Frost, W.; Vallejo-Fernandez, G. The attempt frequency of the antiferromagnetic alloy MnN. *In Preparation* **2024**,
